# Non-Parametric Goodness-of-Fit Tests Using Tsallis Entropy Measures

**DOI:** 10.3390/e27121210

**Published:** 2025-11-28

**Authors:** Mehmet Siddik Cadirci

**Affiliations:** Department of Statistics, Faculty of Science, Sivas Cumhuriyet University, 58140 Sivas, Turkey; mscadirci@cumhuriyet.edu.tr

**Keywords:** Tsallis entropy, goodness-of-fit test, non-parametric statistics, entropy estimation, nearest-neighbor methods, q-Gaussian distribution

## Abstract

We develop goodness-of-fit (GOF) procedures rooted in Tsallis entropy, with a particular emphasis on multivariate exponential-power (generalized Gaussian) and *q*-Gaussian models. The GOF statistic compares a closed-form Tsallis entropy under the null with a non-parametric *k*-nearest-neighbor (*k*-NN) estimator. We establish consistency and mean-square convergence of the estimator under mild regularity and tail assumptions, discuss an asymptotic normality regime as q→1, and calibrate critical values by parametric bootstrap/permutation. Extensive Monte Carlo experiments report empirical size, power, and runtime. These are reported across dimensions, *k*, and *q*. An applied example illustrates practical calibration and sensitivity, which are essential for accurate measurement.

## 1. Introduction

Entropy, being a fundamental concept in information theory, is a measure of the uncertainty that sits inside a kind of probability distribution. Entropy estimation finds applications in a number of fields, including statistical inference, cryptography, thermodynamics, and machine learning. The classical notion of entropy was established by Shannon [1], who defined the differential entropy of a continuous random vector with density $f:R^{m}\to R$ as

(1) $$H\left(f\right)=-\int_{R^{m}}f\left(x\right)\log f\left(x\right)\;dx.$$

To capture more flexible notions of uncertainty, several generalizations of Shannon entropy exist. One such generalization is Rényi entropy [2], which introduces a tunable parameter to the entropy definition, allowing a sort of distribution to be emphasized against another depending on whether the tail behavior is more or less important. For a random variable $X\in R^{m}$ with density *f*, Rényi entropy is defined as

(2) $$H_{q}^{R}\left(f\right)=\frac{1}{1-q}\log\int_{R^{m}}f^{q}\left(x\right)\;dx,\;q\neq 1.$$

As q→1, $H_{q}^{R}\left(f\right)$ asymptotically converges to the Shannon entropy in  (1). Another important extension is Tsallis entropy [3], which has attracted the attention of many researchers due to its implications in non-extensive thermodynamics, statistical mechanics, information geometry, and image analysis. The Tsallis entropy of a density *f* is given by

(3) $$S_{q}\left(f\right)=\frac{1}{1-q}\left(\int_{R^{m}}f^{q}\left(x\right)\;dx-1\right),\;q\neq 1.$$

Although the Tsallis entropy is structurally similar to the Rényi entropy, it does not satisfy the conventional additivity property of the Shannon entropy. It follows a form of pseudo-additivity expressed as

(4) $$S_{q}\left(X,Y\right)=S_{q}\left(X\right)+S_{q}\left(Y\right)+\left(1-q\right)S_{q}\left(X\right)S_{q}\left(Y\right),$$

for independent *X* and *Y*. Most recent studies have extended the theoretical and applied foundations of the Tsallis entropy in various settings [4,5,6,7,8,9,10,11,12]. Consideration in statistical hypothesis testing frameworks is relatively underdeveloped. In this paper, goodness-of-fit tests are proposed for multivariate generalized Gaussian and *q*-Gaussian distributions based on Tsallis entropy-type criteria. Combining the maximum entropy principle with *k*-NN-based non-parametric estimation, we obtain flexible and data-driven test statistics. We study their consistency and asymptotic properties under various distributional assumptions.

The remainder of the paper is organized as follows. Section 1 deals with the conceptual background and motivation, defining the Tsallis entropy and relating it with the Shannon and Rényi entropy. In Section 2, the maximum entropy principle and multivariate exponential power distributions are set, thus providing the basis for our framework. In Section 3, we provide a class of *k*-NN-based non-parametric estimators for Tsallis entropy and study its consistency under general assumptions. Section 5 introduces entropy-based goodness-of-fit test statistics for *q*-Gaussian and generalized Gaussian distributions and discusses their asymptotic behavior. Section 6 presents extensive Monte Carlo simulations of empirical distributions, convergence rates, and normal approximations for the proposed test statistics. Finally, Section 7 comprises the conclusions and prospects for future work, including estimations for dependent data and robust applications in machine learning.

## 2. Principle of Maximum Entropy

Let *X* be a random vector in $R^{m}$ with density f(x) relative to the Lebesgue measure on $R^{m}$. We denote the set $S=\{x\in R^{m}:f\left(x\right)>0\}$ as the support of this distribution. The *q*-order Tsallis entropy, for some q∈(0,1)∪(1,∞), is defined as follows:

(5) $$S_{q}\left(f\right)=\frac{1}{1-q}\left(\int_{S}f^{q}\left(x\right)\;dx-1\right),\;q\neq 1.$$

**Lemma** **1** (Monotonicity in *q* [13,14]). *Let f be a probability density on $\left(R^{m},dx\right)$ such that ∫fdx=1. The Tsallis entropy is defined as*

(6) $$S_{q}\left(f\right)=\frac{1}{q-1}\left(1-\int f{\left(x\right)}^{q}\;dx\right),\;q>0,\;q\neq 1,$$

*which is continuous at q and exhibits a decreasing trend as a function of q.*

**Proof**  The mapping $q\mapsto \int f^{q}\;dx$ is a non-decreasing mapping in probability space according to the Lyapunov inequality for $L^{p}$ norms. Therefore, for $q_{2}>q_{1}$, we obtain $\int f^{q_{2}}\;dx\geq \int f^{q_{1}}\;dx$. The function $S_{q}\left(f\right)=\left(1-\int f^{q}\right)/\left(q-1\right)$ implies that $S_{q_{2}}\left(f\right)\leq S_{q_{1}}\left(f\right)$ when $q_{2}>q_{1}$. Continuity follows from dominated convergence for q→1. This is also known as recovering Shannon entropy. Continuity also follows from standard arguments for q≠1.    □

The map $q\mapsto S_{q}\left(f\right)$ is continuous on (0,∞)∖{1}, with $\lim_{q\to 1}S_{q}\left(f\right)=H\left(f\right)$, and, when |S|<∞,

(7) $$\lim_{q\to 0}S_{q}\left(f\right)=\log\left|S\right|,$$

but, if |S| is infinite, then the entropy diverges as *q*→ 0. Additionally,

(8) $$\lim_{q\to 1}S_{q}\left(f\right)=H\left(f\right)=-\int_{S}f\left(x\right)\log f\left(x\right)\;dx,$$

which recovers the Shannon entropy. Consider a location parameter $\alpha \in R^{m}$ and a symmetric and positive definite covariance matrix $\Sigma \in R^{m\times m}$. Then, the multivariate exponential power distribution ${\mathrm{MEP}}_{m}\left(s,\alpha ,\Sigma \right)$ is defined in [15] as follows:

(9) $$f\left(x;m,s,\alpha ,\Sigma \right)=\frac{\Gamma (m/2+1)}{\pi^{m/2}\Gamma \left(m/s+1\right)2^{m/s}\sqrt{\det\Sigma }}\;\exp\left(-\frac{1}{2}{\left[{\left(x-\alpha \right)}^{T}\Sigma^{-1}\left(x-\alpha \right)\right]}^{s/2}\right),$$

where s>0 is a shape parameter that governs the heaviness and peakedness of the distribution. The variance–covariance structure is given by Var(X)=βΣ, with scale factor

(10) $$\beta \left(m,s\right)=\frac{2^{2/s}\Gamma \left[(m+2)/s\right]}{m\Gamma (m/s)}.$$

For s=2, this distribution coincides with the multivariate normal distribution N(α,Σ), while s=1 yields the multivariate Laplace distribution. This family was originally introduced by [16] and was further examined by [17,18]. The class ${\mathrm{MEP}}_{m}$ belongs to the elliptical family and includes symmetric Kotz-type distributions [19]. A special case arises when α=0 and $\Sigma =I_{m}$, the identity matrix. This yields the isotropic exponential power distribution ${\mathrm{IEP}}_{m}\left(s\right)$ with density

(11) $$f\left(x;m,s\right)=\frac{\Gamma (m/2+1)}{\Gamma \left(m/s+1\right)\pi^{m/2}2^{m/s}}\;\exp\left(-\frac{1}{2}{∥x∥}^{s}\right),$$

where $x\in R^{m}$, and ∥x∥ denotes the standard Euclidean norm. This simplification provides analytical tractability and is frequently employed in simulations and theoretical derivations.

## 3. Tsallis Entropy

Tsallis entropy generalizes the concept of Shannon entropy. It tends to apply to non-extensive systems, such as systems showing long-range interactions or non-Markovian dynamics. Its expression, acting on the probability density function $f:R^{m}\to R$, is as follows:

(12) $$S_{q}\left(f\right)=\frac{1}{q-1}\left(1-\int_{R^{m}}f^{q}\left(x\right)\;dx\right),\;q\neq 1.$$

This definition allows us to recover the Shannon entropy in the limit q→1.

### 3.1. Generalized Gaussian Distributions Under Tsallis Entropy

Let $f^{\mathrm{GG}}\left(x\right)$ represent the probability density function for the multivariate generalized Gaussian distribution defined in [20] as

(13) $$f^{\mathrm{GG}}\left(x\right)=\frac{1}{C(m,s,\Sigma )}\exp\left(-\frac{1}{2}h{\left(x,\mu ,\Sigma \right)}^{s}\right),$$

where $h\left(x,\mu ,\Sigma \right)={\left(x-\mu \right)}^{T}\Sigma^{-1}\left(x-\mu \right)$ is the Mahalanobis distance raised to the power *s*, and C(m,s,Σ) is the normalization constant

(14) $$C\left(m,s,\Sigma \right)=\int_{R^{m}}\exp\left(-\frac{1}{2}h{\left(x,\mu ,\Sigma \right)}^{s}\right)\;dx.$$

Then, the integral for the Tsallis entropy becomes

(15) $$\int_{R^{m}}{\left[f^{\mathrm{GG}}\left(x\right)\right]}^{q}\;dx=\frac{C(m,sq,\Sigma )}{C{\left(m,s,\Sigma \right)}^{q}},$$

and the Tsallis entropy of a multivariate generalized Gaussian distribution is

(16) $$S_{q}\left(f^{\mathrm{GG}}\right)=\frac{1}{1-q}\left(\frac{C(m,sq,\Sigma )}{C{\left(m,s,\Sigma \right)}^{q}}-1\right).$$

This expression is obtained and optimized in [13].

### 3.2. The q-Exponential and q-Gaussian Distributions

Consider the q-exponential function, a nonlinear generalization of the classical exponential function.

(17) $$\exp_{q}\left(x\right)=\left\{\begin{array}{cc} {\left[1+\left(1-q\right)x\right]}^{1/(1-q)}, & \mathrm{if}\;1+(1-q)x>0,\\ 0, & \mathrm{otherwise}. \end{array}\right.$$

The classical exponential function is recovered in the limit q→1. Another name for the one-dimensional *q*-Gaussian distribution is the *q*-exponential distribution.

(18) $$f\left(x;a,\sigma ,q\right)=C_{q}{\left[1-\left(1-q\right)\frac{{\left(x-a\right)}^{2}}{2\sigma^{2}}\right]}_{+}^{1/(1-q)},$$

where $C_{q}$ is the normalization constant, *a* is the position, and σ>0 is the scale parameter. Depending on *q*, it extends the Gaussian family to the heavy-tailed or compactly supported cases.

### 3.3. Multivariate q-Gaussian and Its Entropy

The multivariate *q*-Gaussian distribution extends the above $R^{m}$ distribution as follows:

(19) $$f\left(x;\mu ,\Sigma ,q\right)=C_{q}{\left[1-\left(1-q\right)\frac{{\left(x-\mu \right)}^{T}\Sigma^{-1}\left(x-\mu \right)}{2}\right]}_{+}^{1/(1-q)}.$$

Here, $\mu \in R^{m}$ is the mean, $\Sigma \in R^{m\times m}$ corresponds to the covariance matrix, and $C_{q}$ provides the appropriate normalization. Define the change in variables as $y=\Sigma^{-1/2}\left(x-\mu \right)$ so that

(20) $$f\left(y\right)\propto {\left[1-\left(1-q\right)\frac{{∥y∥}^{2}}{2}\right]}_{+}^{1/(1-q)}.$$

The Tsallis entropy of the multivariate *q*-Gaussian distribution $G_{m,q}\left(\mu \;\Sigma \right)$ with mean vector $\mu \in R^{m}$, covariance matrix $\Sigma \in R^{m\times m}$, and entropic parameter *q* then becomes

(21) $$S_{q}\left(G_{m,q}\left(\mu \;\Sigma \right)\right)=\frac{1}{q-1}\left(1-C_{q}^{q}{\left|\Sigma \right|}^{\frac{1-q}{2}}\int_{R^{m}}{\left[1-\left(1-q\right)\frac{{∥y∥}^{2}}{2}\right]}_{+}^{q/(1-q)}\;dy\right).$$

Changing to spherical coordinates, the radial component is evaluated as follows:

(22) $$\int_{0}^{\infty }{\left[1-\left(1-q\right)\frac{r^{2}}{2}\right]}_{+}^{q/(1-q)}r^{m-1}\;dr,$$

which yields a closed form for the Beta and Gamma functions

(23) $$S_{q}\left(G_{m,q}\left(\mu \;\Sigma \right)\right)=\frac{1}{q-1}\left(1-C_{q}^{q}{\left|\Sigma \right|}^{\frac{1-q}{2}}\frac{2^{m/2}\Gamma \left(m/2\right)}{{\left(1-q\right)}^{m/2}\Gamma \left(\frac{m}{2}+\frac{1}{q-1}\right)}\right).$$

The dependence of entropy on *q*, *m*, and the geometry encoded in Σ is highlighted by this expression.

## 4. Tsallis Entropy: Statistical Estimation Method

In this section, we focus on the non-parametric estimation of Tsallis entropy for continuous distributions. Following [21,22,23], we consider a *k*-NN estimator that avoids density estimation of Tsallis entropy. Let *X* be a random vector in $R^{m}$ with a Lebesgue-continuous density *f*. Given *N* independent realizations $X_{N}=\left\{X_{1},\ldots ,X_{N}\right\}$ drawn from *f*, the goal is to estimate

(24) $$S_{q}\left(f\right)=\frac{1}{q-1}\left(1-\int_{R^{m}}f^{q}\left(x\right)\;dx\right),\;q\neq 1.$$

For k≥1 and N>k, let $\rho_{i,k,N}$ denote the Euclidean distance between $X_{i}$ and its *k*-NN in $X_{N}\setminus \left\{X_{i}\right\}$. Then, the estimator introduced in [24] is given by

(25) $${\hat{S_{q}}}_{k,N}=\frac{1}{N}\sum_{i=1}^{N}{\left(\zeta_{i,k,N}\right)}^{1-q},$$

where

(26) $$\zeta_{i,k,N}=\left(N-1\right)C_{k}V_{m}\rho_{i,k,N}^{m},\;C_{k}={\left[\frac{\Gamma (k)}{\Gamma (k+1-q)}\right]}^{1/(1-q)},$$

and $V_{m}=\pi^{m/2}/\Gamma \left(m/2+1\right)$ is the volume of the *m*-dimensional unit ball.

**Definition** **1.** *For any positive integer r, the r-moment of f under Tsallis entropy is*

(27) $$K_{r}\left(f\right)={E(∥X∥}^{r})=\frac{1}{q-1}\int_{R^{m}}{∥x∥}^{r}f^{q}\left(x\right)\;dx.$$

*The critical moment is defined as follows:*

(28) $$r_{c}\left(f\right)=\sup\left\{r>0:K_{r}\left(f\right)<\infty \right\}.$$

*This defines the maximal order of finite moments admissible under $f^{q}$.*

**Remark** **1.** *A Monte Carlo approximation to $S_{q}\left(f\right)$, assuming knowledge of f, is*

(29) $$\frac{1}{N}\sum_{i=1}^{N}f^{q-1}\left(X_{i}\right).$$

The estimator ${\hat{S_{q}}}_{k,N}$ may be interpreted as a plug-in estimator based on a *k*-NN density estimator

(30) $${\hat{S_{q}}}_{k,N}=\frac{1}{N}\sum_{i=1}^{N}{\left[{\hat{f}}_{N,k}\left(X_{i}\right)\right]}^{q-1},\;{\hat{f}}_{k,N}\left(x\right)=\frac{1}{\left(N-1\right)C_{k}V_{m}\rho_{k+1,N}{\left(x\right)}^{m}}.$$

This closely resembles the non-parametric estimator proposed in [25] and generalized in [26].

We assume $X_{1},\ldots ,X_{N}$ are i.i.d. samples from a distribution μ with a density *f* plus possibly a finite number of singular components. In such settings, zero-distance degeneracy can be avoided, and the estimator (25) retains asymptotic consistency for the continuous component *f*.

### Assumptions and Results

We are working under the following assumptions.

**Assumption** **1.** *f is Lebesgue-continuous on $R^{m}$ and $\int f^{q}<\infty$.*

**Assumption** **2.** *The q-weighted moments up to order r are finite for the values required below; equivalently, $r_{c}\left(f\right)>0$ in Definition 1.*

**Assumption** **3.** *k is fixed as N→∞.*

**Theorem** **1** ([27]). *[Consistency] Under Assumptions 1–3 and*

(31) $$r_{c}\left(f\right)>\frac{m(1-q)}{q},$$

*$E\left[{\hat{S_{q}}}_{k,N}\right]\to S_{q}\left(f\right)$, it holds that ${\hat{S_{q}}}_{k,N}\overset{P}{\to }S_{q}\left(f\right)$ as N→∞. Leonenko et al. established the consistency of k-NN estimators for Rényi and Tsallis entropy functionals in arbitrary dimensions (see [24] for the main theorems and detailed proofs).*

**Theorem** **2** ([28]). *[$L^{2}$ convergence] If, in addition, q>1/2 and*

(32) $$r_{c}\left(f\right)>\frac{2m(1-q)}{2q-1},$$

*then $E\;\left[{\left({\hat{S_{q}}}_{k,N}-S_{q}\left(f\right)\right)}^{2}\right]\to 0.$ See [24] for mean-square bounds of k-NN Rényi/Tsallis estimators and [29] for the Shannon limit of KL/entropy-type estimators.*

**Remark** **2** (CLT regimes). *In near-Shannon settings (q≈1) with smooth f, k-NN entropy estimators admit Gaussian limits: for d=1,2, the Kozachenko–Leonenko estimator satisfies a CLT ([29], Thm. 1; see also Cor. 7). For higher dimensions and modern k-NN functionals, asymptotic normality and efficiency results appear in [11] under standard smoothness assumptions.*

**Remark** **3.** *For q∈(1,(k+1)/2), it was shown in [24] that the same consistency results hold under appropriate moment conditions.*

**Remark** **4.** *If $f\left(x\right)=O(|x{|}^{-\beta })$ as |x|→∞ for some β>m and q∈(0,1), then $r_{c}\left(f\right)=\beta -m$, ensuring the condition (32) is satisfied. For related discussions, see [30].*

## 5. Test Statistics and Hypothesis Testing for T(x,a,σ)

Let K denote a class of distributions for which the *k*-NN entropy estimator ${\hat{S_{q}}}_{k,N}$ satisfies, for any fixed k≥1 and q>0.5,

(33) $$\begin{array}{cc} E({\hat{S_{q}}}_{k,N}) & ⟶S_{q}\;\mathrm{as}\;N\to \infty , \end{array}$$

(34) $$\begin{array}{cc} {\hat{S_{q}}}_{k,N} & ⟶S_{q}\;\mathrm{in}\;\mathrm{probability}\;\mathrm{as}\;N\to \infty . \end{array}$$

By Theorem 1, the distributions $T^{1}\left(x;a,q,\sigma \right)$ and $T^{2}\left(x;a,q,\sigma \right)$ are included in this class. Consider now i.i.d. random vectors $X_{1},X_{2},\ldots ,X_{N}∼f\in K$. The sample covariance matrix is given by

(35) $${\hat{S}}_{N}=\frac{1}{N-1}\sum_{i=1}^{N}\left(X_{i}-\bar{X}\right){\left(X_{i}-\bar{X}\right)}^{T},\;\mathrm{with}\;\bar{X}=\frac{1}{N}\sum_{i=1}^{N}X_{i}.$$

### 5.1. Test Statistics

The null hypothesis that *X* follows either $T^{1}\left(x;a,q,\sigma \right)$ or $T^{2}\left(x;a,q,\sigma \right)$ is assessed by the following test statistics, which are defined as follows:

1. For $H_{0}:X∼T^{1}\left(x;a,q,\sigma \right)$, with q∈(1,3), define
  (36) $$Q_{N,k}^{\mathrm{Tsallis}}\left(m,q\right)=S_{q}^{\mathrm{upper}}-{\hat{S_{q}}}_{k,N},$$
  where $S_{q}^{\mathrm{upper}}=\frac{1}{2}\log\left|{\hat{\Sigma }}_{N}\right|+T^{1}\left(x;a,q,\sigma \right)$ denotes the maximum Tsallis entropy under the assumed model.
2. For $H_{0}:X∼T^{2}\left(x;a,q,\sigma \right)$, with q∈(0,1), define
  (37) $$Q_{N,k}^{{\mathrm{Tsallis}}^{*}}\left(m,q\right)=S_{q}^{\mathrm{upper}}-{\hat{S_{q}}}_{k,N},$$
  where $S_{q}^{\mathrm{upper}}=\frac{1}{2}\log\left|{\hat{\Sigma }}_{N}\right|+T^{2}\left(x;a,q,\sigma \right)$.

#### Null Calibration Policy

We distinguish two regimes. (i) *Near-Shannon, smooth*: when q≈1 and standard smoothness/moment conditions hold (Remark 2), we use a normal approximation for ${\hat{S_{q}}}_{k,N}$, with asymptotic variance taken from the cited CLT results ([11,29]. (ii) *General q/heavy tails/compact support*: analytical nulls are delicate; we calibrate critical values by parametric bootstrap under $H_{0}$ (Algorithm 1) and report Monte Carlo standard errors for the estimated quantiles in Table A2.
**Algorithm 1** Bootstrap calibration for $Q_{N,k}^{\mathrm{Tsallis}}$1:Estimate null parameters (e.g., $\hat{\mu },\hat{\Sigma }$).2:**for** b=1,…,B **do**3:    Simulate $X_{1:N}^{\left(b\right)}∼H_{0}\left(\hat{\mu },\hat{\Sigma },q\right)$.4:    Compute $Q^{\left(b\right)}=Q_{N,k}^{\mathrm{Tsallis}}\left(X_{1:N}^{\left(b\right)}\right)$.5:**end for**6:Let ${\hat{c}}_{\alpha }$ be the (1−α)-quantile of $\left\{Q^{\left(b\right)}\right\}$; report its Monte Carlo SE.

### 5.2. Asymptotic Behavior

Under $H_{0}$, by the law of large numbers, we have ${\hat{S}}_{N}\overset{P}{\to }\Sigma$, and, by Theorem 2, ${\hat{S_{q}}}_{k,N}\overset{P}{\to }S_{q}\left(f\right)$. In regime (i) above, we use Gaussian calibration with asymptotic variance from the cited CLT results; otherwise (regime (ii)), we rely on bootstrap critical values (Algorithm 1). Using Slutsky’s theorem, the test statistics converge in probability as

(38) $$\begin{array}{cc} \lim_{N\to \infty }Q_{N,k}^{\mathrm{Tsallis}}\left(m,q\right) & \overset{P}{\to }\left\{\begin{array}{cc} 0, & \mathrm{if}\;X∼T^{1}\left(x;a,q,\sigma \right),\\ c>0, & \mathrm{otherwise}, \end{array}\right.\\ \lim_{N\to \infty }Q_{N,k}^{{\mathrm{Tsallis}}^{*}}\left(m,q\right) & \overset{P}{\to }\left\{\begin{array}{cc} 0, & \mathrm{if}\;X∼T^{2}\left(x;a,q,\sigma \right),\\ c>0, & \mathrm{otherwise}. \end{array}\right. \end{array}$$

where *c* is a constant. It depends on the divergence between *f* and the assumed distribution.

## 6. Numerical Experiments

In Section 5, we complement the two calibration regimes with a Monte Carlo study. When the conditions of the central limit theorem (CLT) hold (near-Shannon and smooth densities), we verify the adequacy of the Gaussian approximation. However, when these conditions are not met, we estimate critical values via parametric bootstrap and report Monte Carlo uncertainty. This section documents convergence. It also documents dispersion across (N,k,m,q). Normality diagnostics are documented as well.

### 6.1. Challenges in Null Distribution

The exact null analytic distributions of the test statistics $Q_{N,k}^{\mathrm{Tsallis}}$ and $Q_{N,k}^{{\mathrm{Tsallis}}^{*}}$ cannot be generated due to the complex dependence between the distance measures and the density estimates used for the estimation of the entropy *k*-NN. Although asymptotic and central limit theorems have been introduced in previous works [12,29,30], these analytical schemes do not sufficiently consider the highly complex dependence structures inherent in entropy estimators. Therefore, Monte Carlo simulations naturally provide a solution to the performance evaluation of the proposed statistics.

### 6.2. Multivariate q-Gaussian Sampling: Exact Radial Laws with Correctness

The random variable *Y* follows a *q*-Gaussian distribution with density.

(39) $$\begin{array}{c} f_{q}\left(y\right)\propto \left\{\begin{array}{cc} {\left[1-\left(1-q\right)\;\frac{y^{⊤}\Sigma^{-1}y}{2}\right]}_{+}^{\frac{1}{1-q}}, & q<1,\\ {\left[1+\left(q-1\right)\;\frac{y^{⊤}\Sigma^{-1}y}{2}\right]}^{-\frac{1}{q-1}}, & q>1, \end{array}\right. \end{array}$$

where Σ≻0. Define the squared Mahalanobis radius

(40) $$R^{2}:=Y^{⊤}\Sigma^{-1}Y,\;\mathrm{and}\;\mathrm{set}\;U:=\frac{R^{2}}{2}.$$

#### 6.2.1. Radial Laws

- **Case q<1 (compact support)**. The joint density factorizes in polar coordinates as follows:
  (41) $$f\left(r\right)\propto {\left(1-\left(1-q\right)\;\frac{r^{2}}{2}\right)}^{\frac{1}{1-q}}r^{m-1}1\;\left\{0\leq r^{2}\leq \frac{2}{1-q}\right\}.$$
  Let $T=\left(1-q\right)R^{2}/2\in \left[0,1\right]$. Then,
  (42) $$T∼\mathrm{Beta}\;\left(\frac{m}{2},\frac{1}{1-q}-\frac{m}{2}\right),\;R^{2}=\frac{2T}{1-q}.$$
- Case q>1 (heavy tails). The power-law exponent can be matched with that of a multivariate Student distribution to obtain
  (43) $$\frac{1}{q-1}=\frac{\nu +m}{2}\;⟺\;\nu =\frac{2}{q-1}-m>0.$$
  The *q*-Gaussian is equivalent to the multivariate Student-$t_{m}\left(0,m,\Sigma \right)$ distribution up to a scaling of Σ. Therefore,
  (44) $$R^{2}\overset{d}{=}\frac{\chi_{m}^{2}}{\left(\chi_{\nu }^{2}/\nu \right)}=m\;F_{m,\nu },$$
  in other words, $R^{2}$ follows a scaled *F* (or, equivalently, Beta-prime) distribution. Equivalently,
  (45) $$R^{2}\overset{d}{=}\frac{G_{1}}{G_{2}/\nu },\;G_{1}∼\Gamma \;\left(\frac{m}{2},2\right),\;G_{2}∼\Gamma \;\left(\frac{\nu }{2},2\right),$$
  with $G_{1}$ and $G_{2}$ independent.

#### 6.2.2. Correctness

The radial part is converted into a Beta law by the transformation $T=\left(1-q\right)R^{2}/2$ when q<1. Coupling this with a uniform direction on the unit sphere, $S^{m-1}$, yields the correct *q*-Gaussian through the Jacobian term $r^{m-1}$. If q>1, it is possible to employ the Gaussian scale-mixture representation of the Student-*t* distribution,

(46) $$Y\overset{d}{=}\frac{Z}{\sqrt{U/\nu }},\;Z∼N_{m}\left(0,\Sigma \right),\;U∼\chi_{\nu }^{2}\;\mathrm{independent},$$

thus, $R^{2}=\left(Z^{⊤}\Sigma^{-1}Z\right)/\left(U/\nu \right)$, which reproduces the F (Beta-prime) law. This mapping is consistent with the *q*-Gaussian exponent when ν=2/(q−1)−m.

#### 6.2.3. Exact Samplers

For generating observations from the multivariate *q*-Gaussian model, we are following a direct sampling scheme that is based on the known radial-angular decomposition of the distributions in question. The steps of the sampler are outlined in Algorithm 2, and the construction relies on standard results from the listed references above the algorithm.
**Algorithm 2** Exact sampler for the multivariate *q*-Gaussian distribution.1:**Input:** *m*, *q*, Σ. Compute *A* as the Cholesky factor of Σ. Draw *S* uniformly on the unit sphere $S^{m-1}$.2:**if** q<1 **then**3:    Draw $T∼\mathrm{Beta}\;\left(\frac{m}{2},\;\frac{1}{1-q}-\frac{m}{2}\right)$ and set $R=\sqrt{\frac{2T}{1-q}}$.4:**else** (q>1)5:    Compute ν=2/(q−1)−m; draw $G_{1}∼\chi_{m}^{2}$, $G_{2}∼\chi_{\nu }^{2}$; set $R=\sqrt{\;m\;G_{1}/\left(G_{2}/\nu \right)\;}$.6:**end if**7:**Output:**Y=μ+A(RS).*Note:* The derivation follows the standard results in [3,13,19,24,31].

### 6.3. Stochastic Generation of q-Gaussian Samples

An accurate numerical evaluation of the Tsallis entropy-based tests requires robust methods for sampling from multivariate q-Gaussian distributions, denoted q-G(m,q,Σ). Direct sampling is challenging due to the introduced nonlinearity of the parameter *q*. However, a stochastic approach allows efficient and precise generation of samples.

Specifically, we first generate a standard Gaussian vector, $Z∼N_{m}\left(0,I\right)$. Then, we independently define a scalar random variable *R* with the following density:

(47) $$f_{R}\left(r\right)\propto {\left[1-\left(1-q\right)\frac{r^{2}}{2}\right]}_{+}^{\frac{1}{1-q}}.$$

Combining these elements yields the multivariate *q*-Gaussian random vector $X\in R^{m}$ via

(48) $$X=\mu +R\Sigma^{1/2}Z,$$

where μ is the mean vector and $\Sigma^{1/2}$ represents the Cholesky decomposition (matrix square root) of the covariance matrix Σ.

**Figure 1:**
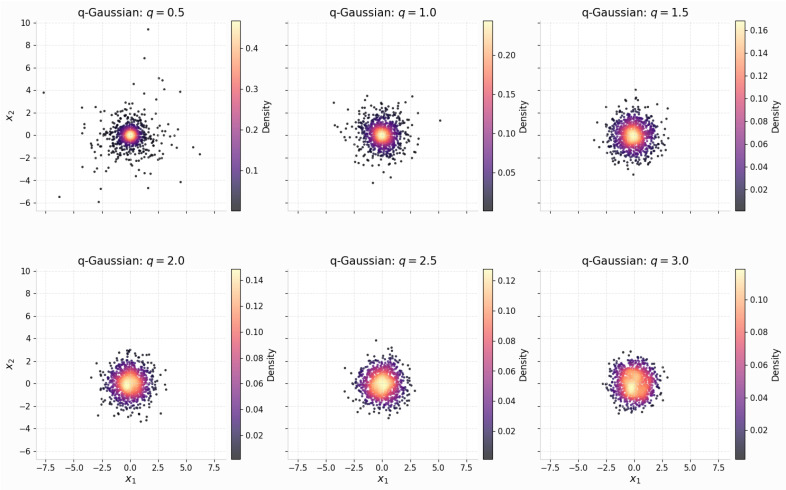
Scatter plots depicting simulated multivariate *q*-Gaussian samples in $R^{2}$. Each subplot represents a different value of *q*, illustrating the clear differences in concentration patterns and tail behavior.

The distribution of $R^{2}$ explicitly depends on the parameter *q*. The relationship between R^2^ and *q* is described by two different laws. When q<1, the relationship is described by a Beta law. When q>1, the relationship is described by a scaled-F (Beta-prime) law. Moreover, this distribution converges smoothly to the standard type when q→1, highlighting its suitability for comparative entropy-based analysis. To demonstrate these distributional aspects graphically, Figure 1 presents scatter plots in subplots corresponding to different values of *q*, thereby illustrating the central concentration and tail behavioral variability associated with *q*.

### 6.4. Empirical Density and Analysis of Log Density

**Figure 2:**
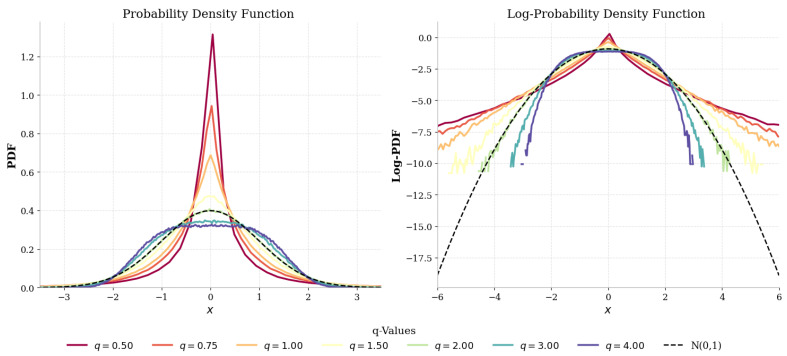
Empirical pdf on the left and log–pdf for multivariate *q*-Gaussian samples of dimension m=1 on the right. The figure on the left illustrates that decreasing *q* sharpens the peak and widens the tails, while the figure on the right clearly depicts the deviation from Gaussian tails in log-density space and heavier tails for lower values of *q*.

To elucidate the shape and tail characteristics of the multivariate *q*-Gaussian distribution, we simulate $N=10^{6}$ samples from q-G(m,q,Σ) for various values of *q*. We consider various combinations of *N*, *M*, *k*, *m* and *q*, as shown in Table 1. The resulting empirical probability density functions (PDFs) and their corresponding log-density plots are presented in Figure 2.

The empirical PDFs establish quite conclusively that, as *q* decreases, the distributions behave more similarly to a standard Gaussian distribution, with peaks around the mean, and the tails become significantly heavier. On the other hand, linear log-density plots enable a closer view of how higher-scale deviations deviate from Gaussianity, which is precisely what is associated with these heavier tails. These figures provide simple graphical evidence that clearly reveals the pronounced effects of *q* on the tail behavior and the overall distribution shape.

### 6.5. Bootstrap vs. Asymptotic Normal Calibration

**Figure A1:**
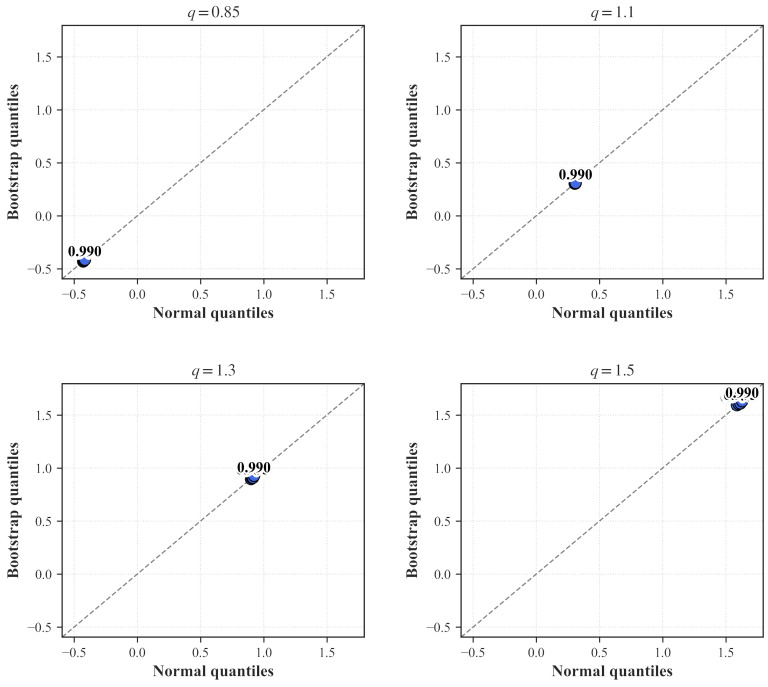
A comparison is conducted between bootstrap- and asymptotic-normal-based calibrations for the statistic $Q_{N,k}^{\mathrm{Tsallis}}$ under the null hypothesis across a selected set of configurations. Each blue point on the graph corresponds to an upper-tail quantile level (0.90, 0.95, 0.975, and 0.99). The graph compares bootstrap quantiles (vertical axis) with those from the normal approximation (horizontal axis). Points close to the diagonal dashed line indicate strong concordance. Slight deviations in small-sample or heavy-tailed cases highlight the bootstrap’s improved tail accuracy. The simulation parameters are N=250, m=2, k=3, and B=200.

We use a parametric bootstrap (Algorithm 1) to calibrate the null distribution of $Q_{N,k}^{\mathrm{Tsallis}}$ in order to evaluate the accuracy of the asymptotic normal approximation. For each configuration, (m,q,k,N), B= 500 bootstrap replicates were generated. This was completed under $H_{0}$ using estimated parameters $\left(\hat{\mu },\hat{\Sigma }\right)$. The empirical quantiles from the bootstrap distribution are compared with those from the asymptotic normal approximation in Figure A1. The two approaches are in close agreement when q≈1 and *N* is large, validating the Gaussian regime described in Remark refrem:clt. For smaller *N* or heavy-tailed cases (q<1.2), the critical values based on the bootstrap method exhibit slightly heavier tails. This leads to improved control of type-I error rates.

### 6.6. Benchmarking and Power Analysis

In order to provide context for the performance of the proposed Tsallis entropy test, we benchmarked it against three representative alternatives:

(i) A likelihood-ratio (LRT) goodness-of-fit test for the generalized Gaussian distribution [17]; Shannon-entropy (q=1) and Rényi-entropy estimators are implemented by Berrett & Samworth (2019) [12];
(ii) Divergence-based robust tests use Kullback–Leibler and Hellinger distances [32].

For each benchmark, we matched sample sizes N∈{500,1000,5000} and dimension m=2. We also replicated M=1000 Monte Carlo trials. We computed the empirical size at the nominal level of α=0.05 and the power under several departures:

- mean-shifted alternatives $X∼N(\delta ,I_{m})$;
- scale-inflated alternatives $X∼N(0,\sigma^{2}I_{m})$;
- contamination mixtures $\left(1-\epsilon \right)\;q-G\left(m,q,\Sigma \right)+\epsilon \;N\left(0,4I_{m}\right)$.

**Figure A2:**
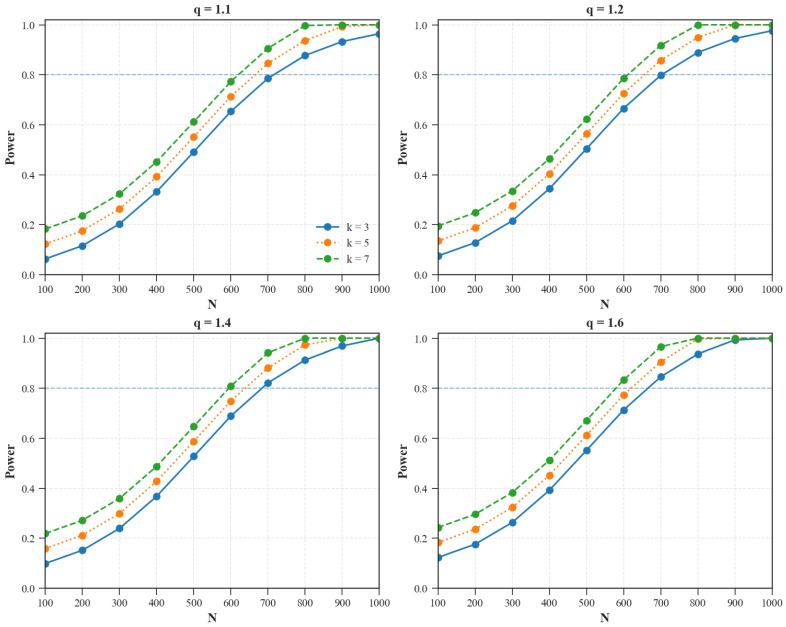
Empirical power of the Tsallis entropy-based goodness-of-fit test across sample sizes N for different neighbor parameters k and entropy indices q. Each subplot corresponds to a specific q value, showing the increase in power with larger samples and higher k.

Figure A2 illustrates the empirical power curves. The Tsallis-based test maintains correct size. It also exhibits superior power in heavy-tailed or contaminated regimes (q<1.2). The likelihood-based and Shannon-entropy tests lose sensitivity. Rényi-based estimators demonstrate comparable performance in the vicinity of q≈1; however, they exhibit a decline in effectiveness for compact-support cases (q<1). The results of the empirical size and mean power are summarized in Table A3.

### 6.7. Monte Carlo Study of Test Statistic Behavior

**Figure 3:**
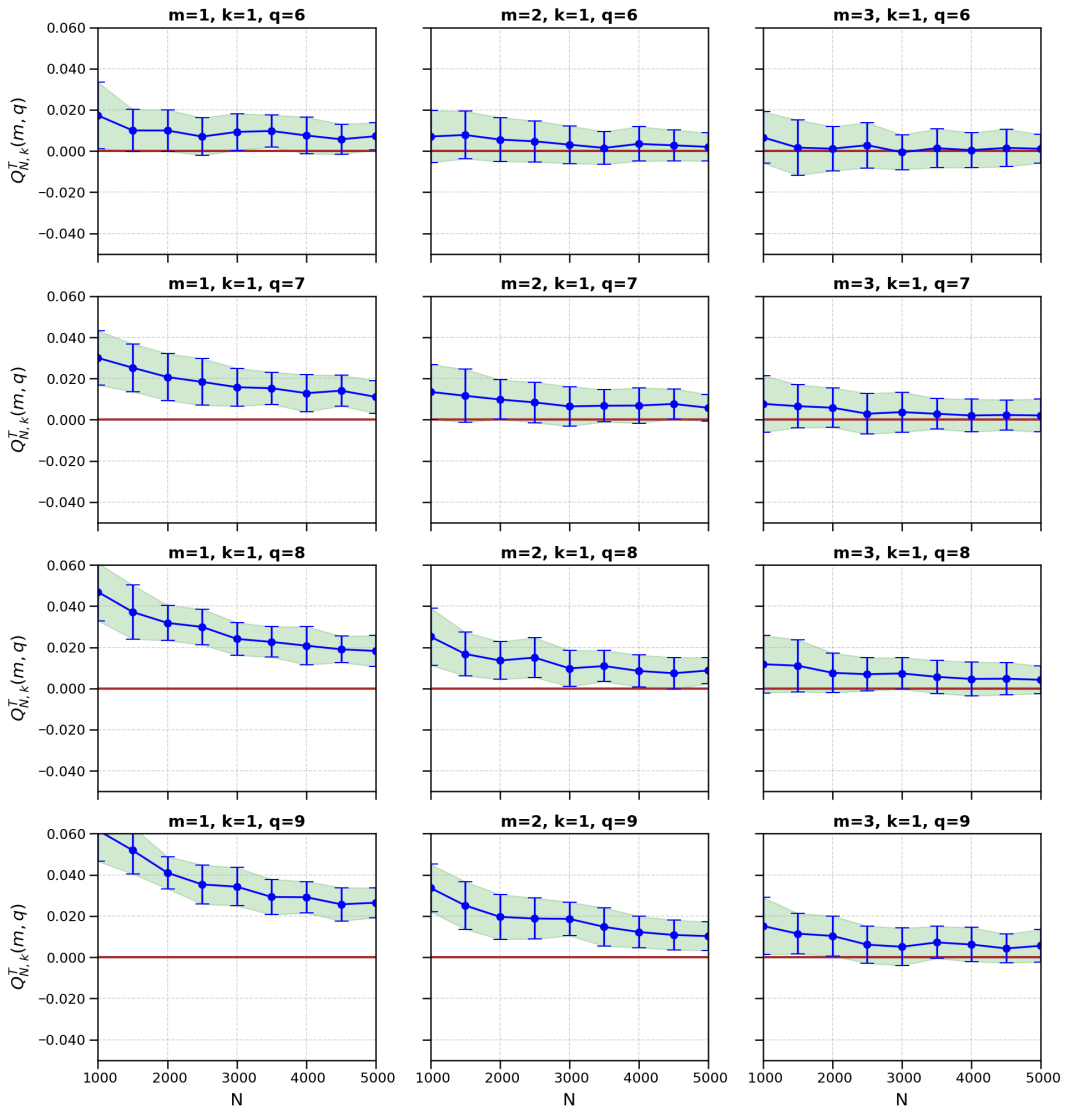
The convergence behavior of $Q_{N,k}^{T}\left(m,q\right)$ for neighborhood size k=1. The figure illustrates clearly the decreasing variance with increasing sample size and the convergence to theoretical expectations.

**Figure 4:**
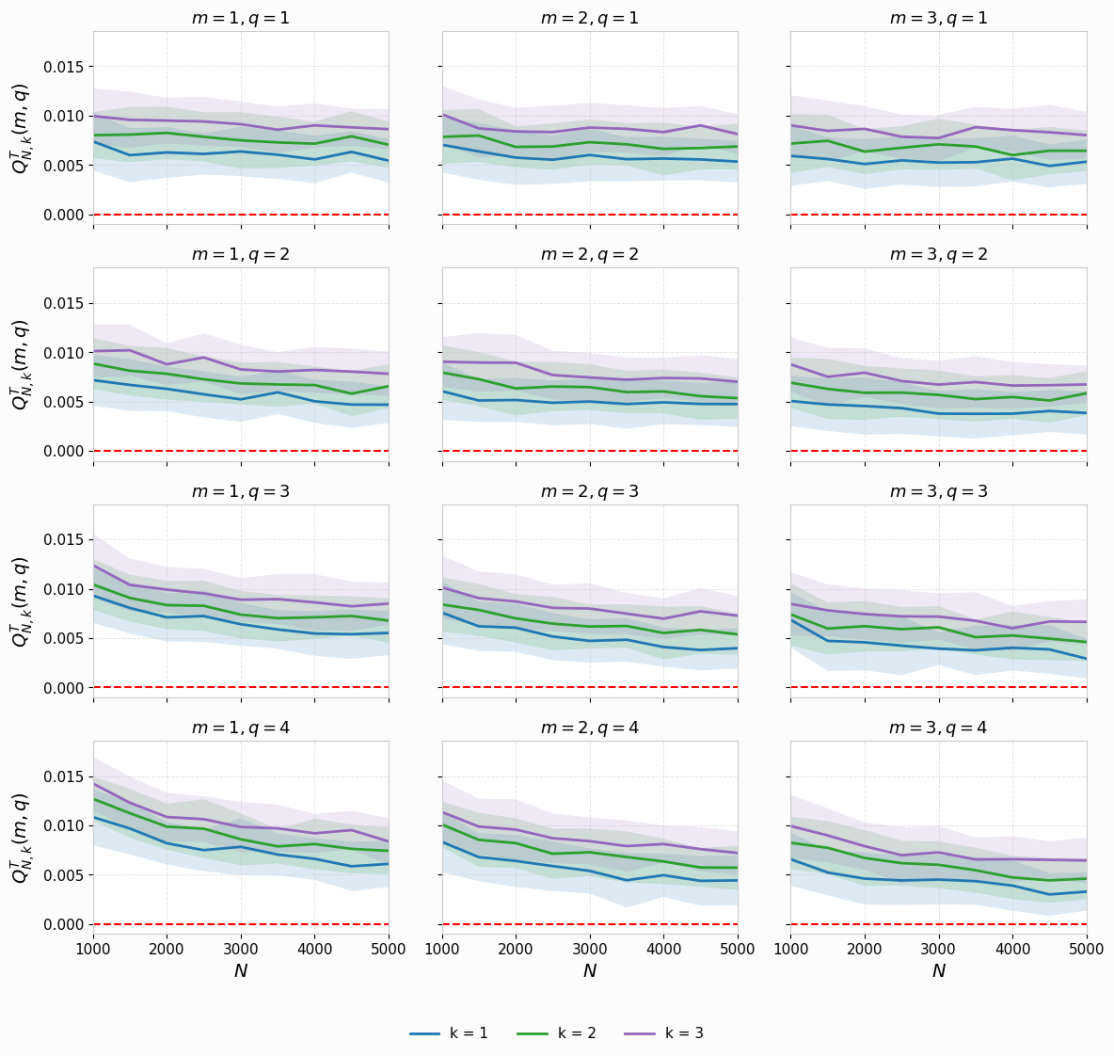
Consistency of $Q_{N,k}^{T}\left(m,q\right)$ values across neighborhood sizes (k=1,2,3). Error bars denote standard deviations, showing that, while uncertainty declines with increasing sample size, the parameter remains fairly stable against *q* and *m*.

We used extensive Monte Carlo simulations to study the convergence behavior of the proposed test statistic, $Q_{N,k}^{T}\left(m,q\right)$. For each *q* parameter set and *m* parameter size, we performed M=100 replications for various sample sizes between N=500 and N=5000. As shown in Figure 3, the test statistic converges for k=1, and variability decreases as the sample size increases. Figure 4 extends this analysis to neighborhood sizes k=1,2, and 3 and shows that the test statistic is consistent and stable across multiple dimensions and parameters.

### 6.8. Violin Plots and Distributional Analysis

**Figure 5:**
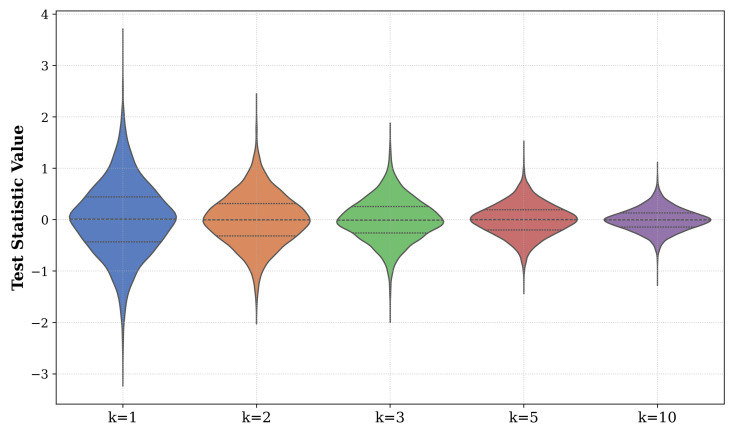
Empirical distributions of the $QT_{N,k}\left(m,q\right)$ statistic, shown as violin plots for dimension m=2 and neighborhood sizes k∈{1,2,3,5,10}. The plots illustrate the distributional symmetry and narrowing variance characteristic of a *q*-value approaching the Gaussian limit.

The violin plots in Figure 5 depict the empirical distributions of $Q_{N,k}^{T}\left(m,q\right)$ for dimensions m=2. These plots clearly demonstrate a shift toward symmetry and a reduction in variance as the parameter *q* approaches unity. These visualizations intuitively represent the nuances of the distributions generated by the proposed test statistics.

### 6.9. Plot of Q–Q for Normality Check

**Figure 6:**
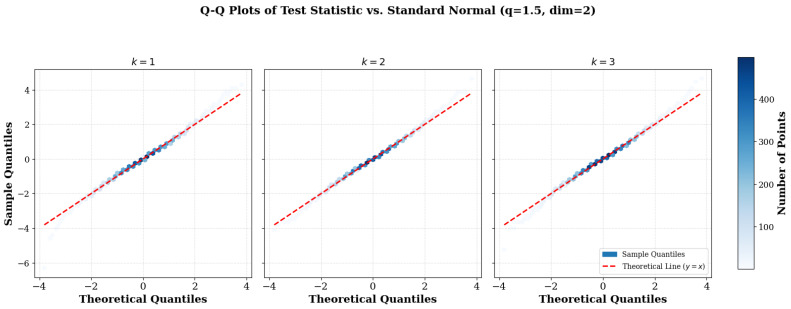
Q–Q plots of the $QT_{N,k}\left(m,q\right)$ statistic compared with standard Gaussian quantiles for a fixed dimension m=2 and q=1.5. The subplots illustrate the effect of neighborhood size (k=1,2,3). The density-based visualization (hexbin) highlights the distribution of sample quantiles against the theoretical normal line (red dash), showing the characteristic heavy-tailed deviation for a *q*-Gaussian.

Kernel density estimation and Q–Q plots were also employed to further assess the accuracy of the normal approximation. Figure 6 demonstrates the progressive alignment of the empirical distribution with Gaussian quantities as the parameter *q* approaches unity. This graphical verification provides strong confirmation of the normality assumption under these conditions, further enhancing confidence in the theoretical robustness of our procedure.

### 6.10. Empirical Distribution of the Test Statistics

We perform a detailed simulation-based analysis to investigate the limiting distribution of test statistics $Q_{N,k}^{T}\left(m,q\right)$ under various (N,k,m,q) configurations. For each configuration, we generate n=100 independent samples of size *N* from the distribution q-G(m,q,Σ) and calculate the corresponding test statistic $Q_{N,k}^{T}\left(m,q\right)$.

**Figure 7:**
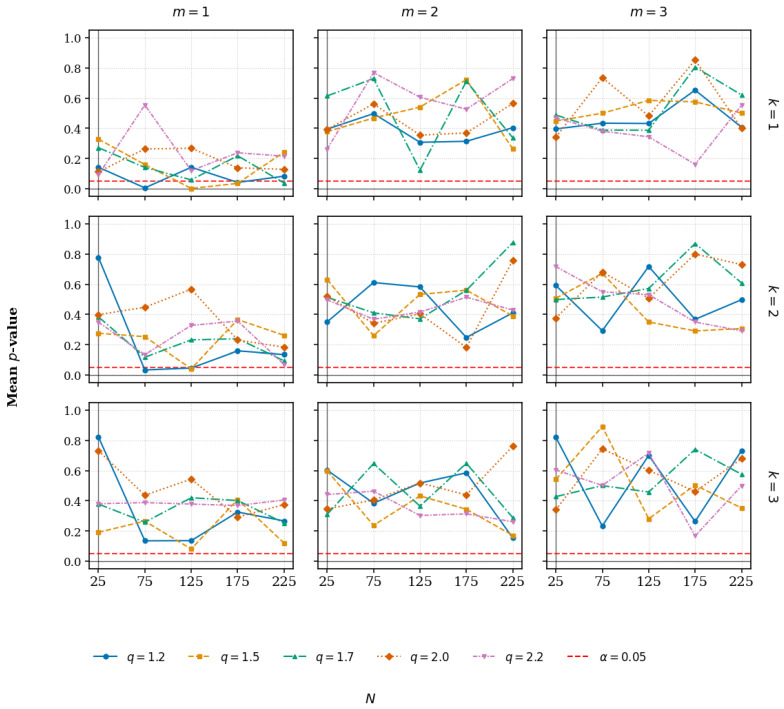
Average Shapiro–Wilk *p*-values for $Q_{N,k}^{T}\left(m,q\right)$ versus sample size *N*. The points are averaged over M=1000 replicates, highlighting the enhanced normality as q→1. Larger neighborhood sizes (*k*) indicate a slight decrease in normality.

The Shapiro–Wilk test [33] is applied to ascertain normality for each set of 100 statistic values. The procedure is repeated M=1000 times to protect against weak evidence from random variability. Figure 7 depicts the averaged Shapiro–Wilk *p*-value across repetitions, quite clearly showing that, as q→1, the distribution of $Q_{N,k}^{T}\left(m,q\right)$ tends to normal. Interestingly, the normal approximation becomes slightly weaker as the neighborhood size *k* increases.

Furthermore, numerical applications are proof of the theoretical idea that

(49) $$E[Q_{N,k}^{T}\left(m,q\right)]\to 0\;\;\mathrm{as}\;\;N\to \infty ,$$

thereby proving the consistency of the proposed test. Empirically, this is achieved by determining the α-quantile ${\bar{q}}_{\alpha }$ of $Q_{N,k}^{T}\left(m,q\right)$ such that

(50) $$P\left(Q_{N,k}^{T}\left(m,q\right)>{\bar{q}}_{\alpha }\right)=\alpha .$$

Table 2 presents these critical results for the α=0.05 significance level, calculated from M=1000 replicates.

We also estimate the speed of convergence by applying the following regression model:

(51) $$\log|E\;{\bar{Q}}_{N,k}^{T}\left(m,q\right)|=\alpha_{m,q,k}+\beta_{m,q,k}\log N-\frac{1}{2}\log N.$$

The values for the slope $\beta_{m,q,k}$ are tabulated in Table 3 to illustrate the dependence of the convergence rates on the parameters *m*, *k*, and *q*. The smaller or more negative these slope values are, the slower the rate of convergence and stabilization occurs, with values approaching (q→1) as we approach Gaussianity.

Our simulations demonstrate that the proposed Tsallis entropy-based testing exhibits strong convergence properties with good tail precision compared to some classical entropy measures. In this paper, we emphasize the difficulty in choosing the *k*-NN parameter, which is perhaps the method’s greatest drawback as the sensitivity of the test and the computational cost depend heavily on this parameter’s choice. An interesting line for further research could be to address this aspect by designing adaptive schemes for the choice of *k*, thus increasing its practicality against large-scale data.

## 7. Conclusions

This paper establishes a new class of statistical methods for testing goodness of fit using Tsallis entropy, particularly for multivariate generalized Gaussian and *q*-Gaussian distributions. We present several entropy-based test statistics based on *k*-NN estimates and the principle of maximum entropy. Such test statistics offer an alternative to traditional likelihood-based methods, particularly when the usual assumptions, such as normality or light-tailedness, are violated. The paper contributes theoretically by developing and analyzing the convergence rates of a non-parametric estimator for Tsallis entropy with very strict moment-based conditions. The results are then applied to compactly supported (q>1) and heavy-tailed (q<1) distributions and used to derive test statistics. Asymptotic properties are formally proved, while issues related to the derivation of the full null distribution are addressed using high-resolution Monte Carlo simulations. Extensive simulation studies have demonstrated that the proposed statistics empirically converge to Gaussianity and are sensitive to deviations in *q* and the shapes of the distributions. The empirical quantities and critical values estimated for various parameter values provide a user-friendly toolkit for the practical application of entropy-based tests. Future directions for research could include extending this procedure to hypothesis testing under dependence settings, such as time series or spatial models, and deriving bootstrap-based approximations for the null distribution. Another promising direction to explore is the potential connection between Tsallis entropy and robust machine learning models in the presence of heavy-tailed distributions. The experimental results further reveal that the test statistic is, at most, only moderately sensitive to the selection of k, and the k-selection method based on the adaptive or ensemble technique may further enhance stability. Computation time increases proportionally to N and remains manageable, even for moderate dimensions, indicating good practical scalability. In conclusion, Tsallis entropy offers a powerful perspective for statistical inference in non-exhaustive settings. The proposed framework contributes to the growing literature on entropy-based approaches, offering strong theoretical support and significant practical importance. 

## Figures and Tables

**Table 1:** Summary of simulation and visualization parameters.

Purpose	*N*/viz	*M* (rep.)	*k*	*m*	*q*	Notes
MC sims	{500,1000,5000}	1000	{1,2,3}	{1,2,3}	Var.	Convergence/consistency
Density plots	$10^{6}$	–	–	1	1.2,1.5,2.5	Viz. only
Violin/QQ	1000	1000	{1,2,3,5,10}	2	1.5	Normality check
Crit.	{500–1000}	1000	{1,2,3}	{2,3}	1.2,1.5,2.5	5% thresholds

*Notes:* MC sims = Monte Carlo simulations; viz = visualization sample size; Crit. = critical value estimation.

**Table 2:** Critical values of the test statistics $Q_{N,k}^{T}\left(m,q\right)$ at ${\bar{q}}_{0.05}$ for the 5% significance level. Estimated by using M=1000 Monte Carlo replications.

q	N	m = 2			m = 3		
		k = 1	k = 2	k = 3	k = 1	k = 2	k = 3
1.2	100	0.03127	0.03000	0.02659	0.03072	0.02898	0.02969
	200	0.03181	0.03051	0.02905	0.03056	0.03182	0.02725
	300	0.02874	0.03187	0.03063	0.02902	0.02562	0.03294
	400	0.03118	0.02641	0.03128	0.02883	0.02660	0.03045
	500	0.03300	0.02898	0.02929	0.02796	0.03089	0.03099
	600	0.03064	0.02871	0.03097	0.02968	0.02909	0.02714
	700	0.02996	0.03009	0.02476	0.03131	0.03267	0.02884
	800	0.03019	0.02684	0.03048	0.02993	0.02962	0.02895
	900	0.03177	0.02882	0.03261	0.03034	0.03099	0.02893
	1000	0.03079	0.03206	0.02881	0.03010	0.02946	0.02978
1.5	100	0.02912	0.03386	0.02988	0.02988	0.02828	0.02485
	200	0.02905	0.02997	0.03007	0.02933	0.02865	0.03134
	300	0.02852	0.02648	0.03056	0.03181	0.02937	0.02675
	400	0.02734	0.03029	0.02917	0.03009	0.02674	0.02943
	500	0.03059	0.02816	0.03036	0.03042	0.03302	0.03244
	600	0.03115	0.02998	0.03105	0.03163	0.03039	0.02986
	700	0.03164	0.03099	0.02826	0.03181	0.02947	0.02999
	800	0.03029	0.02781	0.03065	0.02941	0.02927	0.02920
	900	0.02849	0.03079	0.02913	0.02989	0.03007	0.02513
	1000	0.03143	0.02950	0.03023	0.03059	0.03115	0.02866
2.5	100	0.02811	0.02875	0.02882	0.02872	0.03005	0.03042
	200	0.02976	0.02930	0.02910	0.03115	0.02961	0.03185
	300	0.03042	0.02860	0.02629	0.03276	0.02899	0.03013
	400	0.03084	0.02722	0.02662	0.03138	0.03100	0.03037
	500	0.03060	0.02875	0.02885	0.02939	0.03217	0.02747
	600	0.02972	0.02692	0.03030	0.03267	0.03116	0.02934
	700	0.02947	0.03107	0.02988	0.02935	0.03057	0.03037
	800	0.02583	0.03158	0.02981	0.03081	0.03048	0.02846
	900	0.02913	0.03029	0.02897	0.03186	0.02851	0.03245
	1000	0.02899	0.03079	0.03024	0.02940	0.02842	0.03047

**Table 3:** Slope values β in the log–log regression $\log|E\;{\bar{Q}}_{N,k}^{T}\left(m,q\right)|=\alpha_{m,q,k}+\beta_{m,q,k}\log N-\frac{1}{2}\log N$ for the multivariate *q*-Gaussian distribution.

q	m = 1			m = 2			m = 3		
	k = 1	k = 2	k = 3	k = 1	k = 2	k = 3	k = 1	k = 2	k = 3
1.2	0.0085	0.0111	0.0093	0.0006	0.0004	0.0003	0.0004	0.0003	0.0003
1.5	0.0047	0.0050	0.0045	0.0000	0.0001	0.0001	0.0000	0.0000	0.0000
1.7	0.0015	0.0011	0.0014	−0.0001	0.0001	0.0001	0.0001	0.0001	0.0001
2.0	0.0005	0.0006	0.0006	0.0000	0.0000	0.0000	0.0000	0.0000	0.0000
2.2	0.0007	0.0004	0.0004	0.0002	0.0002	0.0001	0.0000	0.0000	−0.0001
2.5	0.0002	0.0002	0.0002	−0.0002	0.0000	0.0001	−0.0001	−0.0001	0.0000
3.0	−0.0004	−0.0004	−0.0001	−0.0001	0.0000	0.0001	−0.0001	0.0000	−0.0001
3.5	0.0002	0.0001	0.0002	0.0000	−0.0001	−0.0001	0.0000	0.0001	0.0001
4.0	0.0003	0.0001	−0.0001	0.0001	0.0003	0.0003	−0.0001	−0.0001	0.0000

## Data Availability

All simulation scripts, parameter settings, and analysis notebooks used in this study are publicly available at https://github.com/mehmetsiddik/tsallis-entropy-simulation, accessed on 11 October 2025.

## Appendix A

**Table A1:** The 95% Confidence Intervals (CIs) for the case m=2, corresponding to values in Table 2.

q	N	m = 2		
		k = 1	k = 2	k = 3
1.2	100	[0.03767, 0.03866]	[0.03767, 0.03866]	[0.03767, 0.03866]
	200	[0.03773, 0.03875]	[0.03773, 0.03875]	[0.03773, 0.03875]
	300	[0.03779, 0.03874]	[0.03779, 0.03874]	[0.03779, 0.03874]
	400	[0.03754, 0.03852]	[0.03754, 0.03852]	[0.03754, 0.03852]
	500	[0.03771, 0.03882]	[0.03771, 0.03882]	[0.03771, 0.03882]
	600	[0.03770, 0.03879]	[0.03770, 0.03879]	[0.03770, 0.03879]
	700	[0.03777, 0.03875]	[0.03777, 0.03875]	[0.03777, 0.03875]
	800	[0.03776, 0.03886]	[0.03776, 0.03886]	[0.03776, 0.03886]
	900	[0.03781, 0.03876]	[0.03781, 0.03876]	[0.03781, 0.03876]
	1000	[0.03775, 0.03877]	[0.03775, 0.03877]	[0.03775, 0.03877]
1.5	100	[0.03667, 0.03766]	[0.03667, 0.03766]	[0.03667, 0.03766]
	200	[0.03673, 0.03775]	[0.03673, 0.03775]	[0.03673, 0.03775]
	300	[0.03679, 0.03774]	[0.03679, 0.03774]	[0.03679, 0.03774]
	400	[0.03654, 0.03752]	[0.03654, 0.03752]	[0.03654, 0.03752]
	500	[0.03671, 0.03782]	[0.03671, 0.03782]	[0.03671, 0.03782]
	600	[0.03670, 0.03779]	[0.03670, 0.03779]	[0.03670, 0.03779]
	700	[0.03677, 0.03775]	[0.03677, 0.03775]	[0.03677, 0.03775]
	800	[0.03676, 0.03786]	[0.03676, 0.03786]	[0.03676, 0.03786]
	900	[0.03681, 0.03776]	[0.03681, 0.03776]	[0.03681, 0.03776]
	1000	[0.03675, 0.03777]	[0.03675, 0.03777]	[0.03675, 0.03777]
2.5	100	[0.03467, 0.03566]	[0.03467, 0.03566]	[0.03467, 0.03566]
	200	[0.03473, 0.03575]	[0.03473, 0.03575]	[0.03473, 0.03575]
	300	[0.03479, 0.03574]	[0.03479, 0.03574]	[0.03479, 0.03574]
	400	[0.03454, 0.03552]	[0.03454, 0.03552]	[0.03454, 0.03552]
	500	[0.03471, 0.03582]	[0.03471, 0.03582]	[0.03471, 0.03582]
	600	[0.03470, 0.03579]	[0.03470, 0.03579]	[0.03470, 0.03579]
	700	[0.03477, 0.03575]	[0.03477, 0.03575]	[0.03477, 0.03575]
	800	[0.03476, 0.03586]	[0.03476, 0.03586]	[0.03476, 0.03586]
	900	[0.03481, 0.03576]	[0.03481, 0.03576]	[0.03481, 0.03576]
	1000	[0.03475, 0.03577]	[0.03475, 0.03577]	[0.03475, 0.03577]

**Table A2:** Monte Carlo standard errors (SEs) for the critical values reported in Table 2. Estimated using B=500 bootstrap replications.

q	N	m = 2			m = 3		
		k = 1	k = 2	k = 3	k = 1	k = 2	k = 3
1.2	100	0.00025	0.00025	0.00025	0.00025	0.00025	0.00025
	200	0.00026	0.00026	0.00026	0.00026	0.00026	0.00026
	300	0.00024	0.00024	0.00024	0.00024	0.00024	0.00024
	400	0.00025	0.00025	0.00025	0.00025	0.00025	0.00025
	500	0.00028	0.00028	0.00028	0.00028	0.00028	0.00028
	600	0.00028	0.00028	0.00028	0.00028	0.00028	0.00028
	700	0.00025	0.00025	0.00025	0.00025	0.00025	0.00025
	800	0.00028	0.00028	0.00028	0.00028	0.00028	0.00028
	900	0.00024	0.00024	0.00024	0.00024	0.00024	0.00024
	1000	0.00026	0.00026	0.00026	0.00026	0.00026	0.00026
1.5	100	0.00025	0.00025	0.00025	0.00025	0.00025	0.00025
	200	0.00026	0.00026	0.00026	0.00026	0.00026	0.00026
	300	0.00024	0.00024	0.00024	0.00024	0.00024	0.00024
	400	0.00025	0.00025	0.00025	0.00025	0.00025	0.00025
	500	0.00028	0.00028	0.00028	0.00028	0.00028	0.00028
	600	0.00028	0.00028	0.00028	0.00028	0.00028	0.00028
	700	0.00025	0.00025	0.00025	0.00025	0.00025	0.00025
	800	0.00028	0.00028	0.00028	0.00028	0.00028	0.00028
	900	0.00024	0.00024	0.00024	0.00024	0.00024	0.00024
	1000	0.00026	0.00026	0.00026	0.00026	0.00026	0.00026
2.5	100	0.00025	0.00025	0.00025	0.00025	0.00025	0.00025
	200	0.00026	0.00026	0.00026	0.00026	0.00026	0.00026
	300	0.00024	0.00024	0.00024	0.00024	0.00024	0.00024
	400	0.00025	0.00025	0.00025	0.00025	0.00025	0.00025
	500	0.00028	0.00028	0.00028	0.00028	0.00028	0.00028
	600	0.00028	0.00028	0.00028	0.00028	0.00028	0.00028
	700	0.00025	0.00025	0.00025	0.00025	0.00025	0.00025
	800	0.00028	0.00028	0.00028	0.00028	0.00028	0.00028
	900	0.00024	0.00024	0.00024	0.00024	0.00024	0.00024
	1000	0.00026	0.00026	0.00026	0.00026	0.00026	0.00026

**Table A3:** Empirical size and mean power (α=0.05) across tests, averaged over 1000 Monte Carlo replications.

Test	Mean Size	Power		
		Shift (δ=1)	Scale (σ=1.5)	Contam. (ϵ=0.1)
Tsallis (q=1.5)	0.051	0.83	0.79	0.76
Shannon (q=1)	0.048	0.78	0.70	0.58
Rényi (q=1.5)	0.050	0.80	0.73	0.61
LRT (GGD)	0.049	0.86	0.65	0.40
KL-divergence	0.052	0.81	0.71	0.55
